# Case of a Patient Who Voided Hydatides with His Urine; with an Account of the Appearances on Dissection

**Published:** 1781-02

**Authors:** 


### SECTION II.

### ESSAYS AND OBSERVATIONS.

I. *Cafe of a Patient who voided Hydatides with his Urine; with an Account of the Appearances on Diffection. Communicated by* Thomas Blackburne, M. D. *Phyfician at Durham. Read* February 18, 1781.

W. G. aged 32, by trade an upholfterer, of a robuft and healthy conftitution, on the 18th of Auguft 1774, had the misfortune to fall from a table in a room where he was at work, by which he was very much hurt, and particularly complained of fevere pain in his left hypogaftrium, but, as it foon abated, he applied for no relief at that time.

On the 27th of the fame month he was feized in the night with a total fuppreffion of urine, attended with great ficknefs and vomiting, and a violent pain in the region of the left kidney; he was bled, had a turpentine clyfter, and an oily purging medicine which was repeated every hour, and though the greateft part of it was thrown up again, it procured him feveral loofe ftools in the courfe of the day.

On

On the 28th, all his ſymptoms remaining, the ſemicupium was ordered and repeated twice a day, along with clyſters, opiates, oily diuretic medicines, &c. but without effect, till the 6th of September, when finding a ſudden inclination, he called for a baſon, and made near two quarts of coffee-coloured urine, mixed with a conſiderable number of hydatides, ſome whole and ſome burſt, from the ſize of a pin's head to that of a nutmeg. He was immediately relieved from his pain, all the bad ſymptoms diſappeared, and he was able in a few days to return to work at his buſineſs.

After this time, he uſed frequently to paſs hydatides of the ſame ſize, and ſometimes reddiſh ſabulous matter, eſpecially after taking exerciſe on horſeback.

He remained in this ſtate till the 6th of September 1778, when he was again ſeized with a total ſuppreſſion of urine, acute pain in his left ſide, and vomiting, which continued for eight or ten hours, and was relieved by his paſſing an hydatid, which was burſt, but from the appearance of the bag, muſt have been as large as a hen's egg when it was whole. He continued eaſy till the 10th, when he had a ſevere return of his former ſymptoms; ſuppreſſion of urine (though without pain or apparent tenſion of the bladder)

diſtreſſing diſtreſſing nauſea and vomiting, pain in the left hypogaſtrium, pulſe hard and full, heat not much greater than natural; he was bled, had an oily emulſion c. tinct. Theb. three turpentine clyſters in the courſe of the day, and the decoct. nitroſ. c. ſperm. ceti. On the 11th a purging oily mixture was given without effect, was repeated on the 12th, and produced ſeveral copious evacuations; and on the 13th, the chamomile fomentation was applied to the region of the kidney.

Theſe means being unſucceſsful in procuring an evacuation of urine, and the pain in the kidney being now ſo much abated as hardly to be perceived by the patient, an emulſion with 5 grains of camphor, and 15 drops of tinct. cantharidum, was ordered every 4 hours; continued on the 15th, and the doſe of tinct. canthar. increaſed to 30 drops. He remained tolerably eaſy till the 19th, when the pain returned with violence, and he died the ſame evening.

Having procured leave to examine the body, we found, on opening the abdomen, a ſlight appearance of a mortification of the jejunum towards the right ſide, extending about 3 inches in length, and of the under ſurface of the omentum that was in contact with it. We could find no traces of a kidney or ureter on the right ſide.

 The

The left kidney was increaſed uniformly in all its dimenſions to 5 times the uſual ſize; weighed about a pound and a quarter, and extended conſiderably under the ribs into the hypochondrium. The omentum adhered to it on the upper part, and where it adhered was upwards of 2 inches thick.

On ſeparating the omentum from the kidney, we found a large maſs of coagulated blood, which ſeemed to have proceeded from ruptured veſſels in the ſubſtance of the kidney, which was very tender, and in a diſſolved ſtate throughout.

The pelvis of the kidney was greatly enlarged, and contained about half a pint of a thick mucous fluid, and a large triangular ſtone, one angle of which was cloſely preſſed into the paſſage of the ureter, and entirely cloſed it up. A number of ſmall hydatides was alſo found, ſome burſt, ſome containing a thick fluid, and one a ſmall ſtone about the ſize of a pea. The ureter was enlarged to the thickneſs of a man's little finger, and both that and the bladder were quite empty. The other abdominal viſcera were in a perfectly ſound ſtate

